# Rodent model of disuse-induced bone loss by hind limb injection with botulinum toxin A

**DOI:** 10.1016/j.mex.2020.101079

**Published:** 2020-10-03

**Authors:** Mikkel Bo Brent, Andreas Lodberg, Jesper Skovhus Thomsen, Annemarie Brüel

**Affiliations:** Department of Biomedicine, Aarhus University, Denmark

**Keywords:** Bone loss, Osteopenia, Animal model, Disuse, Mechanical unloading

## Abstract

Bone loss materializes rapidly after immobilization or mechanical unloading. Hind limb injection with botulinum toxin A (BTX) is a highly reproducible animal model for disuse-induced bone loss. Here we describe an easy-to-use and enhanced version of the method employing multiple hind limb injections with BTX to induce a pervasive muscle paralysis and thereby disuse of the hind limb. Thirty-six 12–14-week-old female Wistar rats were stratified into three groups: Baseline (Base), Control (Ctrl), and BTX. Disuse was achieved by injecting BTX directly into the right quadriceps femoris muscle, the hamstring muscles, and the posterior calf muscles. The rats were sacrificed after six weeks, and the right rectus femoris muscle and femur were isolated and analyzed. Hind limb disuse resulted in a significant and substantial loss of both muscle mass and bone mass. The loss of bone mass was accompanied by a reduction of trabecular bone mass and a deterioration of the trabecular micro-architecture with a reduction of trabecular thickness and trabecular number compared to Ctrl. In addition, the trabeculae changed from a more plate-like towards a more rod-like shape as indicated by an increase in the structure model index.

• Multiple injections with BTX targeting muscles on both the anterior and posterior thigh and the calf ensure a uniform and pervasive muscle paralysis and hind limb disuse.
• Hind limb injections with BTX results in a substantial loss of muscle and bone mass and deterioration of the trabecular micro-architecture.
• The induction of hind limb disuse with BTX is highly reproducible.

Specifications table

**Table:** 

Subject Area	Medicine and Dentistry
More specific subject area	Animal model of disuse-induced bone loss
Method name	Rat Model of Localized Disuse Induced by the Clostridium botulinum Toxin.
Name and reference of original method	D. Chappard, A. Chennebault, M. Moreau, E. Legrand, M. Audran, and M. F. Basle. Texture Analysis of X-Ray Radiographs Is a More Reliable Descriptor of Bone Loss Than Mineral Content in a Rat Model of Localized Disuse Induced by the Clostridium botulinum Toxin. Bone Vol. 28, No. 1 January 2001:72–79.

## Method details

Several animal models and protocols have been developed to induce bone loss by hind limb disuse or mechanical unloading. The method presented here is an enhanced version of the rat model of localized disuse induced by the Clostridium botulinum toxin developed by Chappard et al. in 2001. The method uses the potent neurotoxin botulinum toxin A (BTX) to induce muscle paralysis and hind limb disuse. Chappard et al. used a single injection of BTX into the quadriceps muscle of the thigh. However, by injecting BTX into both the quadriceps femoris muscle, hamstring muscles, and posterior calf muscles the neurotoxin is more evenly distributed in the hindlimb muscles resulting in a more reliable, pervasive muscle paralysis and thus hind limb immobilization. The hind limb immobilization results in disuse and mechanical unloading that quickly leads to a substantial loss of both muscle and bone mass. The following materials, procedures, and steps can be used to induce hind limb disuse with BTX in rodents.

**Fig. 1:**
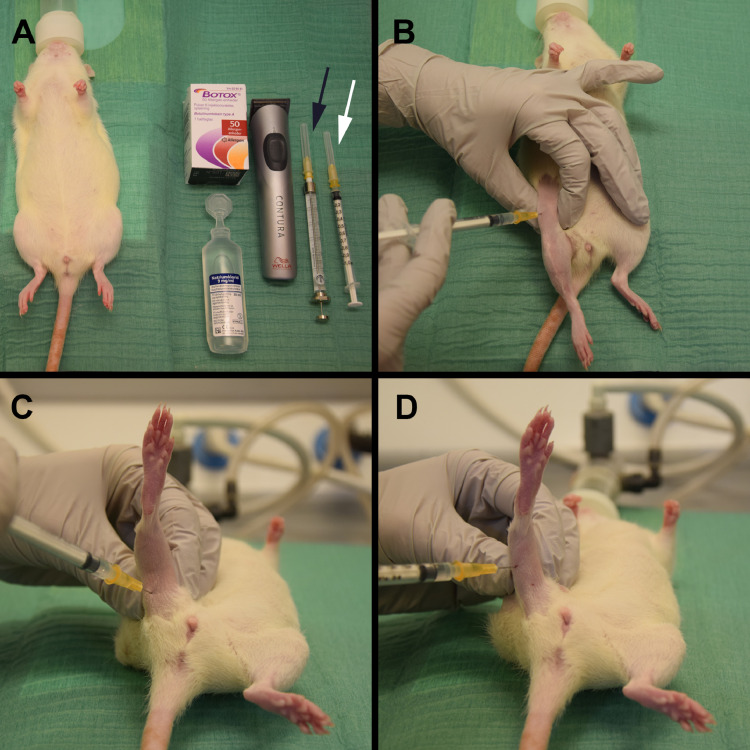
(A) Materials and setup for hind limb injections with saline and BTX. 50 µl Hamilton syringe for mice (black arrow) and 1 ml syringe for rats (white arrow). (B) The right hind limb is shaved and the rectus femoris muscle is exposed using a pincer grasp while BTX is injected. (C-D) The right hip is flexed to expose the calf and hamstring muscles while BTX is injected.

Materials, reagent setup, suggested dose, and safety precautions (Fig. 1)

• Botulinum toxin type A (Botox by Allergan or equivalent)
• Saline (0.9% NaCl)
• 1 ml syringe (to inject rats)
• 50 µl Hamilton syringe (to inject mice)
• 25 gauge (G) needle
• Equipment to induce general anesthesia
• Electric shaver

### Safety precautions when working with BTX

Make sure to read the product information and safety sheet carefully, before working with BTX. Use sufficient personal protective equipment and carry out any procedure involving BTX under a well-ventilated fume hood. BTX is a potent neurotoxin and spread of the toxin can result in swallowing and breathing difficulties, which in serious circumstances may be lethal. Seek immediate medical attention if respiratory, speech or swallowing difficulties occur.

### Procedure

1. Anesthetize the animal with 3% isoflurane by inhalation, and make sure that sufficient general anesthesia is achieved (unresponsiveness to external pain stimuli), before proceeding to the next step.
2. Shave the right hind limb with an electric shaver and remove excess fur.
3. Disinfect the shaved hind limb with 70% alcohol and allow it to dry.
4. Dilute BTX in the vial with saline to a concentration of 20 international units (IU) per ml. For a BTX 50 IU vial add 2.5 ml saline and for a BTX 100 IU vial add 5 ml saline. We suggest a dose of 2 IU per 100 g body weight mice (Table 1) and 4 IU for rats independent of their body weight (Table 2).

**Table 1:** Injection scheme and total diluted BTX (20 IU per ml) volume to be injected (dose of 2 IU per 100 g body weight) tabulated with respect to body weight.

**Mice**	
Injection scheme	
Quadriceps femoris muscle, middle part	½ volume
Calf muscle	½ volume

**Dosing**	
Body weight (g)	Total diluted BTX volume (µl)
15	15
16	16
17	17
18	18
19	19
20	20
21	21
22	22
23	23
24	24
25	25
26	26
27	27
28	28
29	29
30	30

**Table 2:** Injection scheme and total diluted BTX (20 IU per ml) volume to be injected for rats. In contrast to mice, the amount of BTX to be injected in rats (4 IU) is independent of their body weight (12 weeks or older). For younger animals with lower BW dosage should be adjusted accordingly.

**Rats**	
Injection scheme	
Quadriceps femoris muscle: Proximal part	¼ volume
Quadriceps femoris muscle: Distal part	¼ volume
Hamstring muscles	¼ volume
Calf muscle	¼ volume

**Dosing**	
Body weight (g)	Total diluted BTX volume (ml)
All body weights	0.2 (4 IU)
5. For rats (12 weeks or older), use the 1 ml syringe, insert a needle into the vial through the rubber stopper, and pull back on the plunger to withdraw diluted BTX. Put a 25 G needle on the syringe and remove any air bubbles by gently tapping the syringe. Slowly depress the plunger until the needle is filled and the syringe contains the correct amount of diluted BTX. For rats, inject 2 IU BTX into the quadriceps femoris muscle, 1 IU into the hamstrings, and 1 IU into the calf muscles of the right hind limb using the 1 ml syringe (Fig. 1). In practice, hold the muscle group with a firm grip. Insert the needle into the middle of the muscle with the tip pointing in the proximal direction at an angle of 45° to the femoral bone. When the tip touches the bone, withdraw the needle slowly while injecting 0.05 ml into the musculature. While the needle is still just under the skin, repeat the procedure in distal direction. For hamstrings and calf muscles, insert the needle into the middle of the muscles perpendicular to the femur or tibia. When the tip touches the bone, withdraw the needle slowly while injecting 0.05 ml into the musculature.
6. For mice, use a 50 µl Hamilton syringe equipped with a 25G needle. Remove the plunger, and fill the Hamilton syringe and the needle with diluted BTX from the end, where the plunger was removed. When the syringe is completely filled, insert the plunger. Depress the plunger until the syringe contains the correct amount of diluted BTX. Inject half the volume of BTX into the quadriceps femoris muscle and the other half into the calf muscles using the Hamilton syringe. In practice, hold the muscle group with a firm grip. Insert the needle into the middle part of the muscles perpendicular to the femur or tibia. When the tip touches the bone, withdraw the needle slowly while injecting half of the volume into the musculature.
7. Let the animal recover from the anesthetics in an empty housing container and monitor the recovery closely. Normal gait and limb function are conspicuous effected one day after the injections.
8. Neutralize the BTX vial with 0.5% hypochlorite and dispose as hazardous waste.
9. Disposable single-use syringes, which have been in contact with BTX, should be disposed as hazardous waste.
10. Neutralize the Hamilton syringe by filling it with 0.5% hypochlorite and place it horizontally for 5 min. Submerge the plunger in 0.5% hypochlorite for 5 min.
11. Clean the syringe and plunger in sterile H_2_O and thereafter with acetone. Let the syringe and plunger dry, and store for later use.

The Botox injection into the quadriceps femoris muscle mainly targets the rectus femoris and vastus lateralis, medialis and intermedius muscles innervated by the femoral nerve, whereas the injection into the hamstring muscles mainly targets the semimembranosus, semitendinosus and biceps femoris muscles innervated by the tibial part of the sciatic nerve and the common peroneal nerve. Finally, the BTX injection into the calf muscles mainly targets the gastrocnemius and soleus muscle as well as the deep flexor muscles innervated by the tibial nerve. However, it is possible that the injected BTX diffuse and affects other muscles in close proximity to the injection site.

## Method validation

### Animals

Thirty-six 12–14-week-old female Wistar rats (Taconic, Ejby, Denmark) were stratified into the following groups based on their body weight so the average body weight was as similar as possible in the groups: Baseline (Base), Control (Ctrl), and BTX. The Base group was sacrificed at the study start. Hind limb disuse was achieved by injecting BTX directly into the right hind limb musculature as described in detail under the section Method details. Ctrl rats were injected in a similar way with saline only. The rats were housed at 21 °C with 30% humidity, a 12/12-hour light/dark cycle, and free access to tap water and chow (Altromin 1324 Maintenance diet for rats and mice, Brogaarden, Lynge, Denmark). After six weeks, the rats were sacrificed by an overdose of 200 mg/kg pentobarbital i.p. (Mebumal, SAD, Copenhagen, Denmark) under general anesthesia with 3% isoflurane (IsoFlo Vet, Orion Pharma Animal Health, Nivå, Denmark). The right rectus femoris muscle and femur were isolated and stored in Ringers’ solution at −29 °C for the subsequent analyses. The study complied with the guiding principles of the European Communities Council Directive of 24 November 1986 (86/609/EEC) and was approved by the Danish Animal Experiments Inspectorate (2012-15-2934-00,769).

### Data and statistics

The data presented in the section Method validation has previously been partly described [1]. New figures and previously unpublished data on muscle cross-sectional area, cortical tissue mineral density, metaphyseal trabecular volumetric bone mineral density, metaphyseal tissue mineral density, micro-architectural properties of the femoral neck, and femoral mechanical stiffness have been included to support and validate the described method of hind limb disuse with BTX. All data shown in graphs and tables are presented as mean ± standard deviation (SD). The data were analyzed using a one-way ANOVA followed by a post-hoc Holm-Sidak test, whenever Gaussian distribution requirements were met. In the case of a non-Gaussian distribution, a non-parametric one-way ANOVA on ranks followed by a post-hoc Dunn's test was used. Statistical analysis and graphs were made using GraphPad Prism 8.1.1 (GraphPad Software, San Diego, CA, USA).

### Assessment of gait ability

**Fig. 2:**
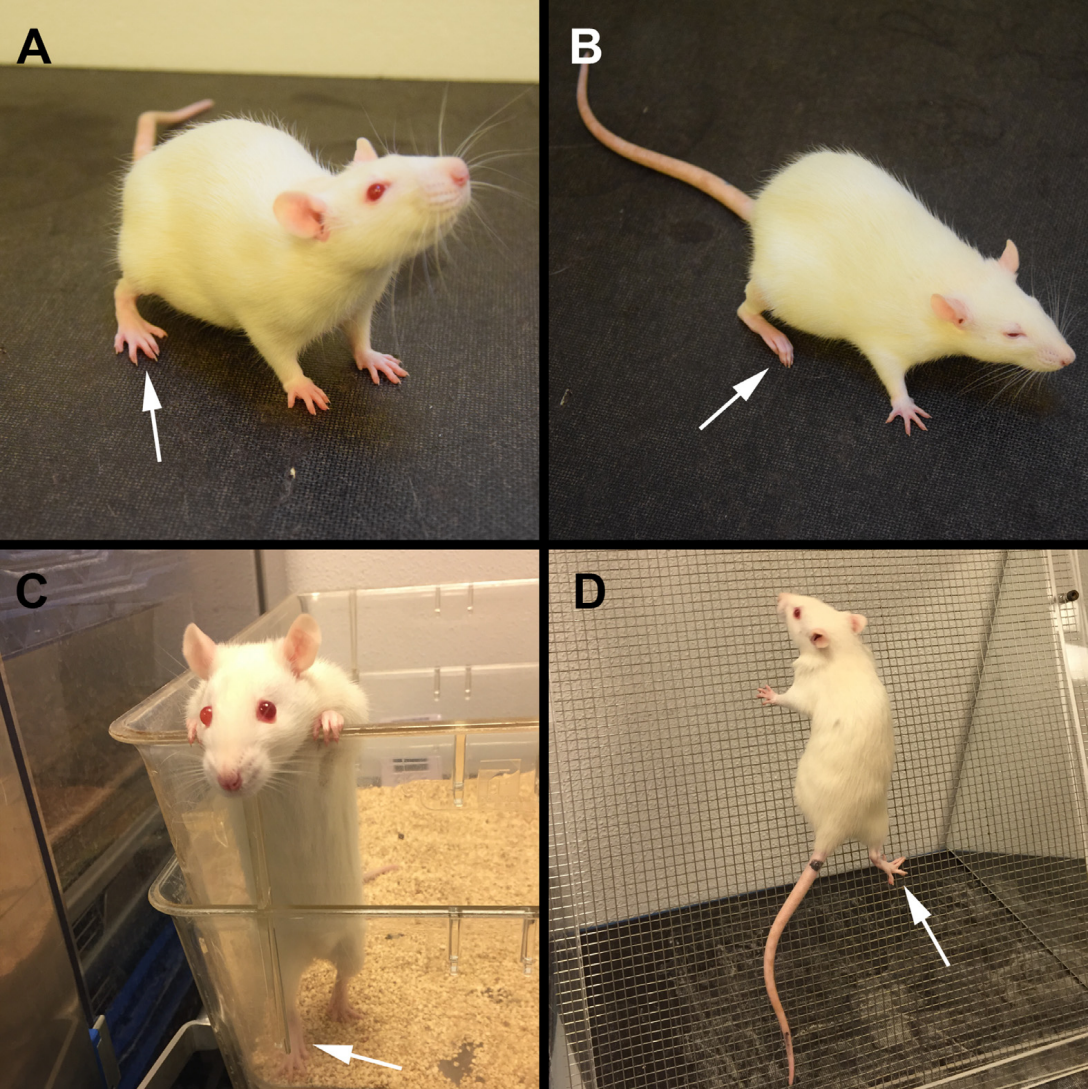
Assessment of gait ability using the score by Warner et al. [2]. (A) Normal toe extension during sitting after saline injections into the right hind limb. (B) Inability to perform toe extension during sitting after injections with BTX into the right hind limb. (C) Normal use and weight bearing of right hind limb during two-legged stance in a rat injected with saline into the right hind limb. (D) Normal use of right hind limb during climbing in a rat injected with saline into the right hind limb. See Video 1–2 in Supplementary materials for assessment of hind limb during level walking. Assessment of hind limb abduction during tail suspension is not shown. White arrow = right hind limb (injected with BTX).

To assess the physiological effect of BTX injections and the degree of hind limb disuse the gait ability score developed by Warner et al. was used [2]. The gait ability score assesses five observations related to voluntary skeletal muscle contractions and movement of the BTX-injected hind limb. 1. Hind limb abduction during tail suspension; 2. Toe extension during sitting; 3. Use of hind limb during level walking; 4. Use of hind limb during two-legged stance; and 5. Use of hind limb during climbing (Fig. 2 and supplementary material Video 1–2). The gait ability score ranged from 0 (completely disabled) to 10 (normal). Six rats from each group were assed upon arrival to our animal facility and weekly during the study with the gait ability score. Injections with BTX resulted in a rapid reduction of free voluntary movement and normal gait ability.

The gait ability score decreased to 0 after the BTX injection and stayed at this level for the following two weeks. Hereafter, the gait ability score slowly increased and reached 9.5 at the end of the study.

### Rectus femoris muscle

**Fig. 3:**
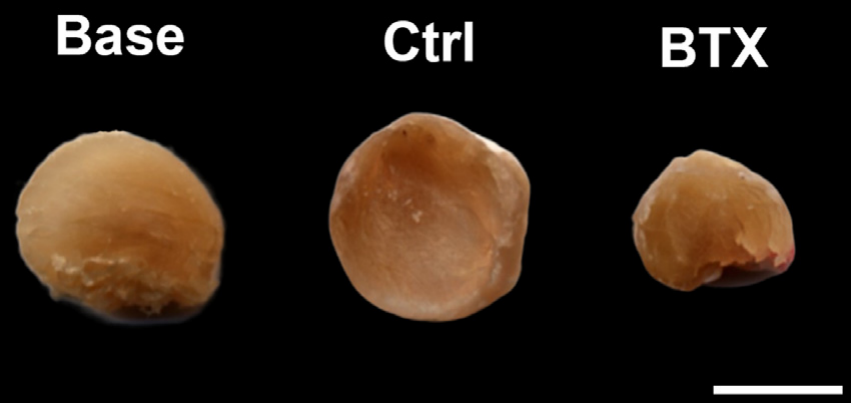
Cross-sectional area of the right rectus femoris muscle from rats injected with saline (Ctrl) or BTX. Bar = 0.5 cm.

The rectus femoris muscle was isolated from the right hind limb and the muscle mass was determined using a digital scale (Mettler AT250, Columbus, OH, USA). The rectus femoris muscle was then halved at the midpoint, placed with the cut surface on a flat-bed image scanner (Perfection 3200 Photo; Seiko Epson, Nagano, Japan), and scanned at a resolution of 3200 DPI using SilverFast SE (version 6.0.1r14; LaserSoft Imaging, Kiel, Germany). The cross-sectional area (CSA) of the cut surface was determined using PhotoShop (version CC2019; Adobe, San José, CA, USA). Finally, the halved rectus femoris muscle was immersion-fixed in 0.1 M sodium phosphate-buffered formaldehyde (4% formaldehyde, pH 7.0) and embedded in plastic based on 2-hydroxyethyl methacrylate (Technovit 7100, Heraeus Kulzer, Wehrheim, Germany). The embedded muscles were cut into 2-µm-thick sections on a microtome (Jung RM2065; Leica Instruments, Nussloch, Germany) and stained with Masson's trichrome. The sections were scanned in a digital slide scanner (NanoZoomer-XR, Hamamatsu, Hamamatsu City, Shizuoka, Japan) and analyzed using newCAST's (Version 6.4.1.2240, Visiopharm, Hørsholm, Denmark) build-in tool to determine striated muscle cell cross-sectional area (Fig. 3).

**Fig. 4:**
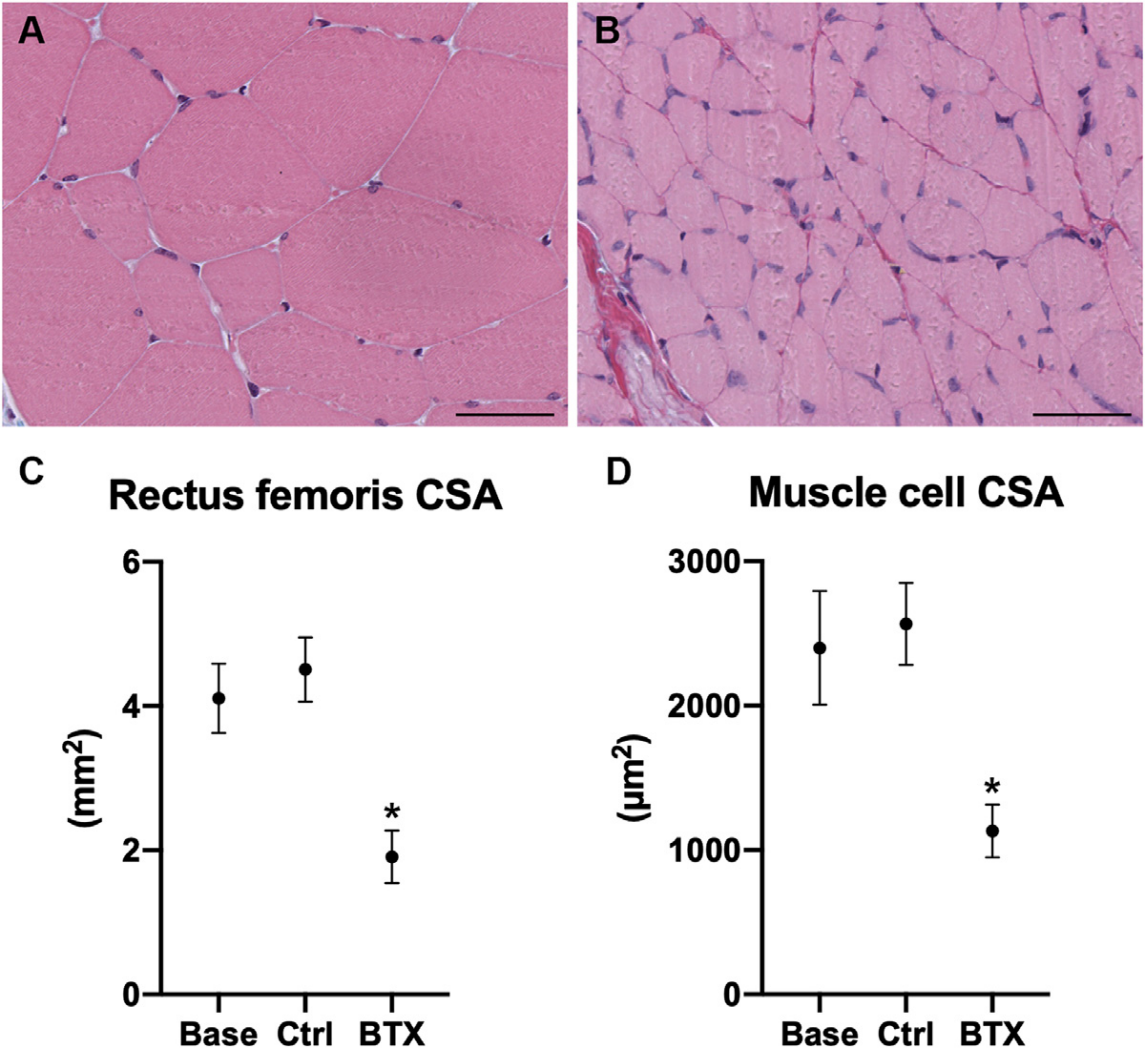
(A) Representative cross-section of the right rectus femoris muscle cells from a non-immobilized rat. (B) Representative cross-section of the right rectus femoris muscle from a rat immobilized with BTX. Bar = 50 µm and stained with Masson's trichrome. (C) Rectus femoris cross-sectional area. (D) Rectus femoris muscle cell cross-sectional area. Data are presented as mean ± SD. * Significant difference (*p* < 0.05) between Ctrl and BTX.

BTX-induced disuse resulted in a significant and substantial loss of rectus femoris muscle mass (−64%) and striated muscle cell cross-sectional area (−56%) compared to Ctrl (Table 3 and Fig. 4).

**Table 3:** Initial and final body weight (BW). Distal femoral metaphyseal volumetric bone mineral density (vBMD), trabecular number (Tb.N), structure model index (SMI), and tissue mineral density (TMD). Femoral mid-diaphyseal cross sectional bone area (B.Ar), marrow area (Ma.Ar), tissue area (T.Ar = *B*.Ar + Ma.Ar), cortical thickness (Ct.Th), TMD, mechanical stiffness ascertained by a three-point bending test. Femoral neck trabecular bone volume fraction (BV/TV), vBMD, trabecular thickness (Tb.Th), trabecular separation (Tb.Sp), Tb.N, connectivity density (CD), SMI, TMD, cortical bone T.Ar, B.Ar, mechanical stiffness ascertained by a femoral neck test. * Statistically significant difference (*p* < 0.05) between Ctrl and BTX. Data are presented as mean ± SD.

	Base	Ctrl	BTX
Initial BW (g)	226 ± 9	226 ± 8	229 ± 10
Final BW (g)	226 ± 9	244 ± 12	221 ± 9*
Rectus femoris muscle mass (mg)	605 ± 52	682 ± 59	248 ± 37*
**Distal femoral metaphysis**			
vBMD (mg/cm^3^)	486 ± 70	463 ± 66	359 ± 57*
Tb.N (mm^−1^)	6.61 ± 0.13	5.65 ± 0.12	4.97 ± 0.10*
SMI (-)	−0.8 ± 1.0	−0.3 ± 0.9	0.9 ± 0.6*
TMD (mg/cm^3^)	907 ± 16	924 ± 19	902 ± 17*
**Femoral mid-diaphysis**			
B.Ar (mm^2^)	4.89 ± 0.26	5.49 ± 0.022	5.03 ± 0.22*
Ma.Ar (mm^2^)	2.65 ± 0.33	2.51 ± 0.50	2.83 ± 0.39
T.Ar (mm^2^)	7.53 ± 0.41	8.00 ± 0.81	7.86 ± 0.54
Ct.Th (mm)	0.60 ± 0.023	0.69 ± 0.032	0.62 ± 0.026*
TMD (mg/cm^3^)	1124 ± 14.4	1160 ± 11.6	1150 ± 12.4
Stiffness (N/mm)	303 ± 32.4	367 ± 42.5	344 ± 36.0
**Femoral neck**			
BV/TV (%)	72.9 ± 7.16	69.8 ± 8.33	52.6 ± 4.40*
vBMD (mg/cm^3^)	763 ± 65.4	750 ± 75.3	601 ± 35.2*
Tb.Th (µm)	121 ± 14.4	117 ± 9.53	97.6 ± 7.95*
Tb.Sp (µm)	90.4 ± 17.5	99.8 ± 22.6	140 ± 15.2*
Tb.N (mm^−1^)	7.40 ± 0.82	7.36 ± 1.06	6.23 ± 0.76
CD (mm^−3^)	125 ± 28.7	118 ± 28.8	122 ± 33.5
SMI (-)	−2.76 ± 0.78	−2.11 ± 0.63	−0.47 ± 0.52*
TMD (mg/cm^3^)	976 ± 18.9	987 ± 22.9	954 ± 16.5*
TA (mm)	4.15 ± 0.67	4.02 ± 1.30	3.61 ± 0.72
BA (mm)	3.02 ± 0.32	3.12 ± 0.93	2.85 ± 0.47
Stiffness (N/mm)	168 ± 35.9	274 ± 79.2	218 ± 57.2*

### Cortical and trabecular bone

**Fig. 5:**
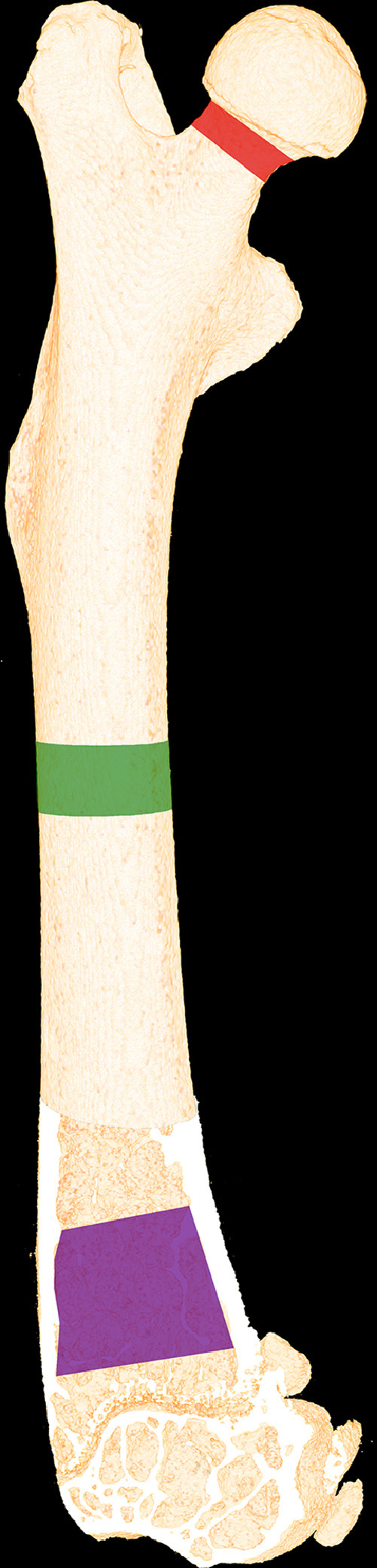
Micro-computed tomography (µCT) scan of femur. The red area represents analyzed volume of interest (VOI) of the femoral neck. The green area represents the VOI consisting of cortical bone only of the femoral mid-diaphysis. The purple area represents the VOI consisting of trabecular bone only of the distal femoral metaphysis. The VOIs are schematic and not to scale.

The right femur was DXA scanned (sabre XL, Nordland Stratec, Pfortzheim, Germany) at a scan speed of 10 mm/s and an isotropic pixel size of 0.5 mm. In addition, the femoral metaphysis, epiphysis, and mid-diaphysis were scanned in a desktop µCT scanner (µCT 35, Scanco Medical AG, Brüttiselen, Switzerland) with an isotopic voxel size of 10 µm, an X-ray tube voltage of 70 kVp and current of 114 µA, an integration time of 800 ms, and 1000 projections/180°. A 0.5 mm aluminum filter was used to reduce beam-hardening effects. The metaphysis was analyzed using a 2200-µm-high volume of interest (VOI) starting 1500 µm below the most distal part of the growth zone in order to exclude primary spongiosa and include trabecular bone only. The epiphysis was analyzed using a VOI starting where the lateral end medial epicondyle fused into one coherent structure and ended where the growth plate first appeared, thus including trabecular bone only. The mid-diaphysis was analyzed using a 2300-µm-high VOI centered on the mid-point of the femur. The femoral neck was aligned parallel with the *y*-axis using IPL (version 5.15, Scanco Medical AG, Brüttiselen, Switzerland) and analyzed using two concentric 600-µm-thick VOIs starting at the subcapital region Fig. 5). One VOI included trabecular bone only, while the other included both trabecular and cortical bone. The 3D data sets were low-pass-filtered using a Gaussian filter (σ = 0.8, support = 1) and segmented with a fixed threshold filter of either 535 mg HA/cm^3^ (distal femoral metaphysis and neck) or 573 mg HA/cm^3^ (femoral diaphysis). Analysis of trabecular bone included: volumetric bone mineral density (vBMD), trabecular thickness (Tb.Th), trabecular number (Tb.N), trabecular separation (Tb.Sp), structure model index (SMI), connectivity density (CD), and tissue mineral density (TMD). Analysis of cortical bone included: cross sectional bone area (B.Ar), marrow area (Ma.Ar), tissue area (T.Ar = B.Ar + Ma.Ar), cortical thickness (Ct.Th), and TMD [3].

**Fig. 6:**
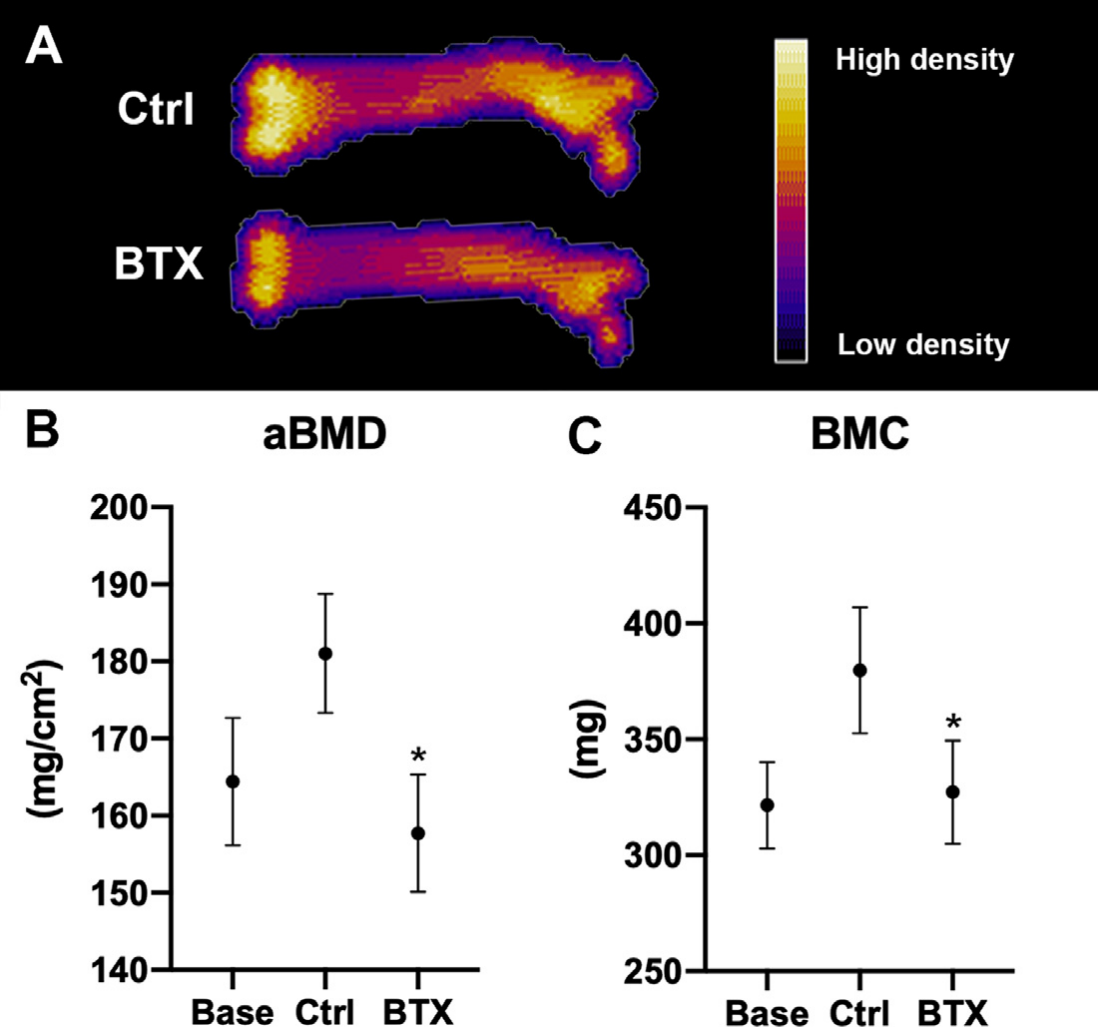
(A) DXA scanning of right femur from Ctrl and BTX. Yellow color represents high bone mineral density, whereas purple color represents low bone mineral density. (B) Bone mineral density (aBMD). (C) Bone mineral content (BMC). Data are presented as mean ± SD. * Significant difference (*p* < 0.05) between Ctrl and BTX.

**Fig. 7:**
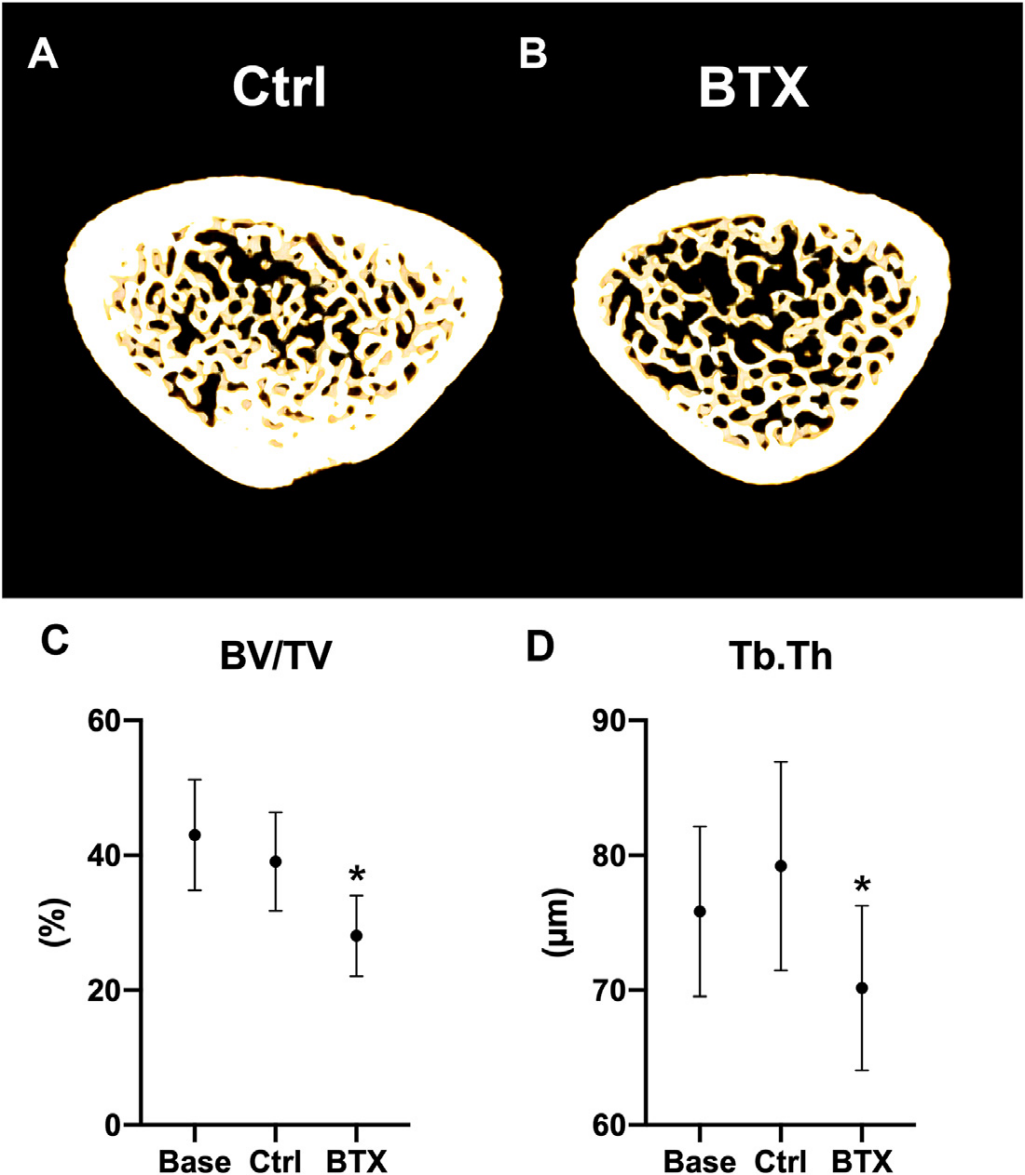
(A, B) Representative three-dimensional images of a 150-µm-thick slice through the distal femoral metaphysis. Note that the immobilization-induced loss of BV/TV and reduction in trabecular number is clearly visible. (C) Bone volume/tissue volume (BV/TV). (D) Trabecular thickness (Tb.Th). Data are presented as mean ± SD. * Significant difference (*p* < 0.05) between Ctrl and BTX.

Disuse resulted in a significant reduction of whole femoral bone mineral density (−13%) and bone mineral content (−14%) compared to Ctrl (Fig. 6). At the femoral metaphysis, disuse significantly reduced bone volume/tissue volume (−28%), volumetric bone mineral density (−22%), trabecular thickness (−11%), trabecular number (−12%), and tissue mineral density (−2%) compared to Ctrl. In addition, the trabeculae changed from a more plate-like towards a more rod-like shape as indicated by an increased SMI (+424%) compared to Ctrl. At the femoral mid-diaphysis, disuse resulted in a significant reduction of cortical bone area (−8%) and cortical thickness (−10%) compared to Ctrl. At the femoral neck disuse significantly reduced volumetric bone mineral density (−20%) bone volume/tissue volume (−25%), trabecular thickness (−17%), tissue mineral density (−3%), increased Tb.Sp (40%), and SMI by 78% (Table 3 and Fig. 7).

### Mechanical testing

**Fig. 8:**
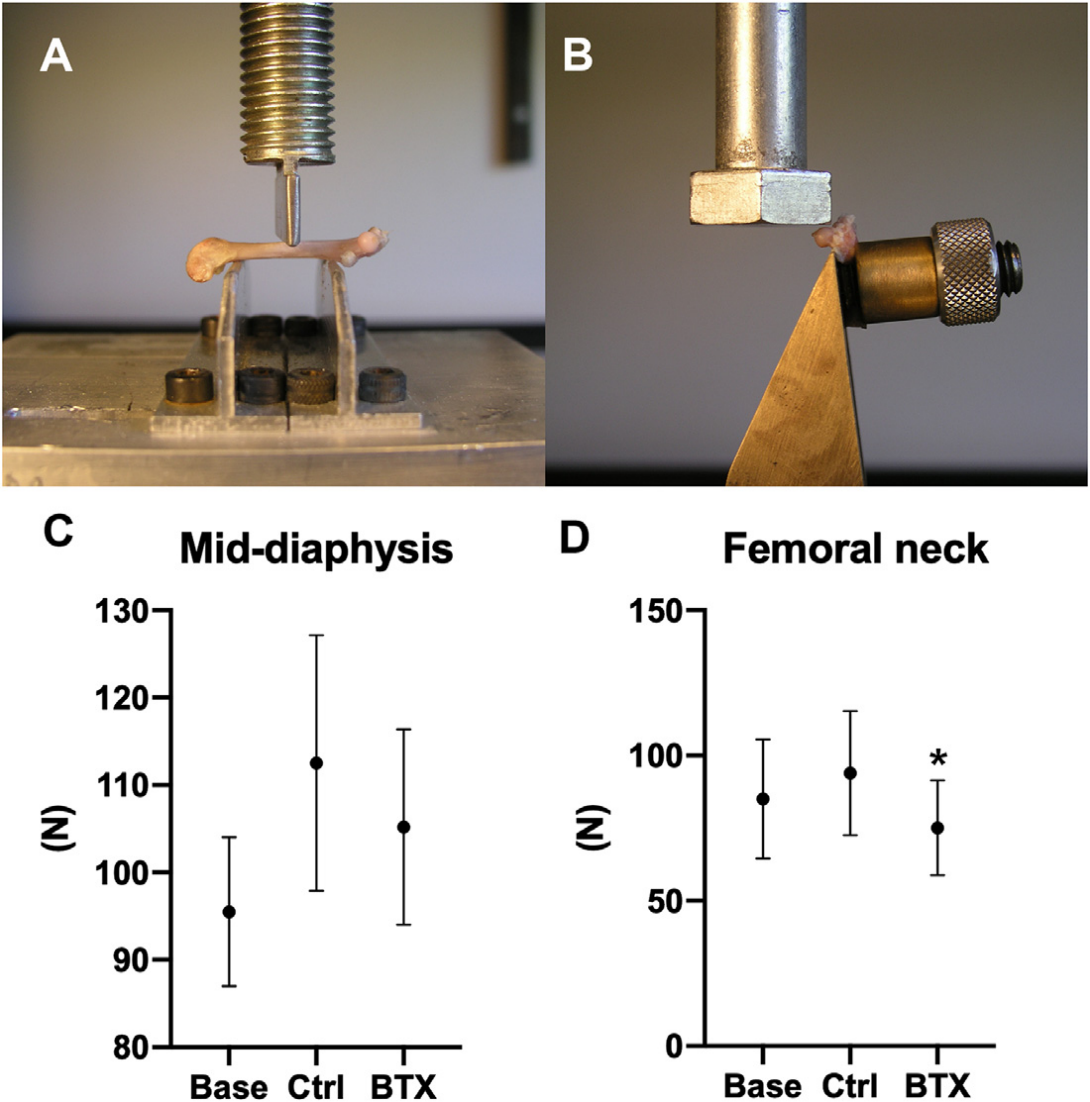
(A) Three-point bending test of the femoral mid-diaphysis and (B) femoral neck test. The load to fracture of the (C) femoral mid-diaphysis and (D) femoral neck.

Load to fracture and maximum stiffness of the right femoral mid-diaphysis and femoral neck were determined using a materials-testing machine (5566; Instron, High Wycombe, UK) [4]. Load was applied with a constant deflection rate of 2 mm/min and load-deformation data were recorded using Merlin (version 3.21, Instron). Disuse resulted in a significant reduction of both load to failure (bone strength) and maximum stiffness (−20% and −20%, respectively) at the femoral neck. Disuse did not significantly reduce bone strength and stiffness at the femoral mid-diaphysis (Fig. 8).

## Additional information

### Mechanism of action of botulinum toxin

**Fig. 9:**
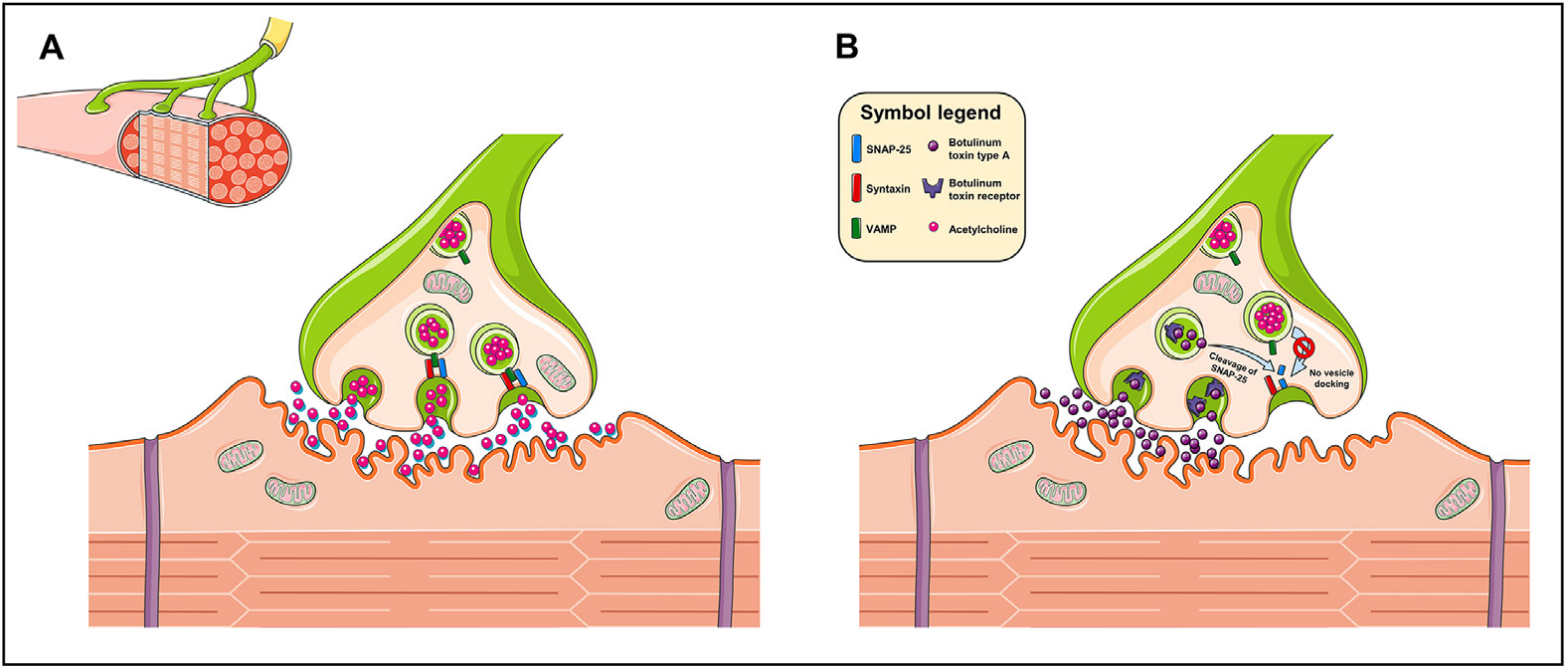
Simplified mechanism of action of botulinum toxin type A. A) Normal release of acetylcholine at the neuromuscular junction. Acetylcholine vesicles migrate to the ending of the axon where the synaptic vesicle-associated membrane protein (VAMP) interacts with the presynaptic synaptosomal-associated protein 25 (SNAP-25) and syntaxin to form the soluble N-ethylmaleimide-sensitive fusion protein attachment protein (SNARE) complex. The SNARE complex allows fusion of the acetylcholine containing vesicles and the presynaptic axon membrane resulting in release of acetylcholine into the neuromuscular junction. B) Botulinum toxin type A is brought into the axon by receptor-mediated endocytosis. The light chain of botulinum toxin type A is translocated from vesicles to the cytosol where it cleaves SNAP-25 preventing formation of the SNARE complex and release of acetylcholine into the neuromuscular junction. Created with images from Servier Medical Art (https://smart.servier.com/) under the Creative Commons License attribution 3.0 Unported License.

Botulinum toxin is the most potent neurotoxin known and is produced by the Gram-positive Clostridium botulinum bacteria [5]. Eight types of botulinum toxins (A-H) have been described [6], but only type A and B are routinely used and approved for use in humans for various dystonic diseases [7,8]. The molecular mechanism of action is through inhibition of motor neuron release of acetylcholine. Upon injection of botulinum toxin into the muscle, the toxin is rapidly internalized in the motor endplates of the neuron by receptor-mediated endocytosis and is stored in vesicles [9]. Translocation of toxin from the vesicles into the cytosol is mediated by acidification of the vesicular lumen resulting in a release of the light chain of the toxin into the cytosol. The light chain of the botulinum toxin types B, D, F, and G are specific proteases cleaving vesicle-associated membrane protein (VAMP), whereas toxin types A, C, and D cleave synaptosomal-associated protein (SNAP-25) (Fig. 9). In addition, toxin type C also cleaves syntaxin [10,11]. VAMP, SNAP-25, and syntaxin are all key proteins in forming the attachment protein receptor (SNARE) complex allowing synaptic vesicle exocytosis of acetylcholine [12]. A more detailed description of the intracellular mechanism of action of botulinum toxins can be found elsewhere [13].

### Dose, number of subsequent BTX injections and study duration

Several protocols have been used to establish a loss of muscle and bone mass by hind limb disuse as a result of botulinum toxin injections in rodents [1,2,[14], [15], [16], [17], [18]]. Different doses, injections sites, and study durations have been used, but no study has systematically compared the different protocols. Several studies have used a BTX dose of 1–2 IU/100 g body weight in both rats and mice. Since the effect of BTX is transient, one could speculate that re-injecting the animal with a second dose, after the effects of the initial dose have worn off, could increase the loss of muscle mass and bone mass. However, we have previously investigated the effect of BTX injections administered at two different time points (first injection at day zero and second injection after four weeks) in rats [19]. We found no further loss of rectus femoris muscle mass, femoral bone mineral density, femoral metaphyseal bone volume/tissue volume, or femoral trabecular thickness after the rats had received the second injection of BTX despite a renewed reduction in gait ability [19]. In addition, we also found no significant difference in bone mineral density, bone volume fraction and femoral neck strength between four and eight weeks of disuse.

Different study durations have been used for mice and rats subjected to disuse by injections with BTX. Hind limb disuse studies in mice are generally shorter in duration than studies in rats. In a long-term study (28 weeks) in rats subjected to hind limb disuse with BTX, we have previously reported the loss of muscle mass, bone mass, and bone strength is maximized after four to eight weeks [20]. In rats subjected to hind limb disuse by injections with BTX, we have previously shown the loss of muscle mass, bone mineral content, trabecular microstructure, and bone strength are increasing week by week after immobilization until the mice were sacrificed after four weeks [4]. However, after three weeks of hind limb disuse, most of the negative effects of immobilization on muscle and bone tissue stagnated.

In summary, we suggest a BTX dose of 2 IU per 100 g body weight for mice and 4 IU for rats regardless of the body weight injected only at the study start, and a study duration of three weeks for mice and four to six weeks for rats to achieve maximized muscle and bone loss.

## Declaration of Competing Interest

The authors declare that they have no known competing financial interests or personal relationships that could have appeared to influence the work reported in this paper.

## Appendix Supplementary materials

Image, video 1

Image, video 2

## References

[1] Brent M.B., Brüel A., Thomsen J.S. (2018). PTH (1–34) and growth hormone in prevention of disuse osteopenia and sarcopenia in rats. Bone.

[2] Warner S.E., Sanford D.A., Becker B.A., Bain S.D., Srinivasan S., Gross T.S. (2006). Botox induced muscle paralysis rapidly degrades bone. Bone.

[3] Bouxsein M.L., Boyd S.K., Christiansen B.A., Guldberg R.E., Jepsen K.J., Müller R. (2010). Guidelines for assessment of bone microstructure in rodents using micro-computed tomography. J. Bone Miner. Res..

[4] Thomsen J.S., Christensen L.L., Vegger J.B., Nyengaard J.R., Brüel A. (2012). Loss of bone strength is dependent on skeletal site in disuse osteoporosis in rats. Calcif. Tissue Int..

[5] Sugiyama H. (1980). Clostridium botulinum neurotoxin. Microbiol. Rev..

[6] Barash J.R., Arnon S.S. (2014). A novel strain of clostridium botulinum that produces type B and type H botulinum toxins. J. Infect. Dis..

[7] Bentivoglio A.R., Del Grande A., Petracca M., Ialongo T., Ricciardi L. (2015). Clinical differences between botulinum neurotoxin type A and B. Toxiconology.

[8] Hallett M., Albanese A., Dressler D., Segal K.R., Simpson D.M., Truong D., Jankovic J. (2013). Evidence-based review and assessment of botulinum neurotoxin for the treatment of movement disorders. Toxiconology.

[9] Montecucco C., Molgó J. (2005). Botulinal neurotoxins: revival of an old killer. Curr. Opin. Pharmacol..

[10] Schiavo G., Matteoli M., Montecucco C. (2000). Neurotoxins affecting neuroexocytosis. Physiol. Rev..

[11] Lalli G., Bohnert S., Deinhardt K., Verastegui C., Schiavo G. (2003). The journey of tetanus and botulinum neurotoxins in neurons. Trends Microbiol..

[12] Ramakrishnan N.A., Drescher M.J., Drescher D.G. (2012). The SNARE complex in neuronal and sensory cells. Mol. Cell. Neurosci..

[13] Zhang S., Masuyer G., Zhang J., Shen Y., Henriksson L., Miyashita S.I., Martínez-Carranza M., Dong M., Stenmark P. (2017). Identification and characterization of a novel botulinum neurotoxin. Nat. Commun..

[14] Chappard D., Chennebault A., Moreau M., Legrand E., Audran M., Basle M.F. (2001). Texture analysis of X-ray radiographs is a more reliable descriptor of bone loss than mineral content in a rat model of localized disuse induced by the Clostridium botulinum toxin. Bone.

[15] Agholme F., Isaksson H., Kuhstoss S., Aspenberg P. (2011). The effects of Dickkopf-1 antibody on metaphyseal bone and implant fixation under different loading conditions. Bone.

[16] Grimston S.K., Silva M.J., Civitelli R. (2007). Bone loss after temporarily induced muscle paralysis by Botox is not fully recovered after 12 weeks. Ann. N. Y. Acad. Sci..

[17] Poliachik S.L., Bain S.D., Threet D.W., Huber P., Gross T.S. (2010). Transient muscle paralysis disrupts bone homeostasis by rapid degradation of bone morphology. Bone.

[18] Manske S.L., Boyd S.K., Zernicke R.F. (2010). Muscle and bone follow similar temporal patterns of recovery from muscle-induced disuse due to botulinum toxin injection. Bone.

[19] Vegger J.B., Brüel A., Thomsen J.S. (2015). Vertical trabeculae are thinned more than horizontal trabeculae in skeletal-unloaded rats. Calcif. Tissue Int..

[20] Bach-Gansmo F.L., Wittig N.K., Brüel A., Thomsen J.S., Birkedal H. (2016). Immobilization and long-term recovery results in large changes in bone structure and strength but no corresponding alterations of osteocyte lacunar properties. Bone.

